# Quercetin prevents necroptosis of oligodendrocytes by inhibiting macrophages/microglia polarization to M1 phenotype after spinal cord injury in rats

**DOI:** 10.1186/s12974-019-1613-2

**Published:** 2019-11-07

**Authors:** Hong Fan, Hai-Bin Tang, Le-Qun Shan, Shi-Chang Liu, Da-Geng Huang, Xun Chen, Zhe Chen, Ming Yang, Xin-Hua Yin, Hao Yang, Ding-Jun Hao

**Affiliations:** 1 Shaanxi Spine Medicine Research Center, Translational Medicine Center, Department of Spine Surgery, Hong Hui Hospital, Xi’an Jiaotong University, 555 You Yi Dong Road, Xi’an, 710054 Shaanxi China; 2 Institute of Neurosciences, Fourth Military Medical University, Xi’an, 710032 Shaanxi China; 3 Department of Laboratory Medicine, Xi’an Central Hospital, Xi’an Jiaotong University, 161 Xi Wu Road, Xi’an, 710003 Shaanxi China; 4 Department of Bone Microsurgery, Hong Hui Hospital, Xi’an Jiaotong University, 555 You Yi Dong Road, Xi’an, 710054 Shaanxi China

**Keywords:** Spinal cord injury, Oligodendrocyte, Necroptosis, Macrophages/microglia, Quercetin

## Abstract

**Background:**

Oligodendrocytes (OLs) death after spinal cord injury (SCI) contributes to demyelination, even leading to a permanent neurological deficit. Besides apoptosis, our previous study demonstrated that OLs underwent receptor-interacting serine-threonine kinase 3(RIP3)/mixed lineage kinase domain-like protein (MLKL)-mediated necroptosis. Considering that necroptosis is always accompanied with pro-inflammatory response and quercetin has long been used as anti-inflammatory agent, in the present study we investigated whether quercetin could inhibit necroptosis of OLs and suppress the M1 macrophages/microglia-mediated immune response after SCI as well as the possible mechanism.

**Methods:**

In this study, we applied quercetin, an important flavonoid component of various herbs, to treat rats with SCI and rats injected with saline were employed as the control group. Locomotor functional recovery was evaluated using Basso-Beattie-Bresnahan (BBB) scoring and rump-height Index (RHI) assay. In vivo, the necroptosis, apoptosis, and regeneration of OLs were detected by immunohistochemistry, 5′-bromo-2′-deoxyuridine (BrdU) incorporation. The loss of myelin and axons after SCI were evaluated by Luxol fast blue (LFB) staining, immunohistochemistry, and electron microscopic study. The polarization of macrophages/microglia after SCI and the underlying mechanisms were detected by quantitative reverse transcription-polymerase chain reaction (qRT-PCR) and immunohistochemistry. In vitro, the ATP and reactive oxygen species (ROS) level examination, propidium iodide (PI) labeling, and Western blotting were used to analyze the necroptosis of cultured OLs, while the signaling pathways-mediated polarization of cultured macrophages/microglia was detected by qRT-PCR and Western blotting.

**Results:**

We demonstrated that quercetin treatment improved functional recovery in rats after SCI. We then found that quercetin significantly reduced necroptosis of OLs after SCI without influencing apoptosis and regeneration of OLs. Meanwhile, myelin loss and axon loss were also significantly reduced in quercetin-treated rats, as compared to SCI + saline control. Further, we revealed that quercetin could suppress macrophages/microglia polarized to M1 phenotype through inhibition of STAT1 and NF-κB pathway in vivo and in vitro, which contributes to the decreased necroptosis of OLs.

**Conclusions:**

Quercetin treatment alleviated necroptosis of OLs partially by inhibiting M1 macrophages/microglia polarization after SCI. Our findings suggest that necroptosis of OLs may be a potential therapeutic target for clinical SCI.

## Background

The secondary injury after spinal cord injury (SCI), more tightly related to severity of neurological deficit, includes cell death, demyelination, axonal degeneration, and inflammatory response [1–3]. Among these, oligodendrocytes (OLs) death after SCI contributes to demyelination of spared axons, even leading to a permanent neurological deficit [4].

Given that the survival of OLs is crucial for functional recovery after SCI, in the present study we focused on death and regeneration of OLs. Apoptosis of OLs occurs mainly in the first several hours after SCI, and the injury is accompanied by chronic inflammatory demyelination [5–7], indicating other types of cell death accounted for the delayed loss of OLs. It has been reported that necroptosis of OLs mediates axonal degeneration in ALS [8]. Our previous study demonstrated that some OLs were receptor-interacting serine-threonine kinase 3(RIP3)/mixed lineage kinase domain-like protein (MLKL)-positive after SCI, indicating that necroptosis occurred in OLs [9]. The chronic, detrimental, M1 macrophages/microglia-mediated inflammatory response contributed to necroptosis of astrocytes after SCI [10, 11]. Likewise, the dominance of M1 sub-population might play a role in necroptosis of OLs. Thus, there is an urgent need for new treatment strategies to prevent OLs death and to ameliorate the M1 macrophages/microglia-mediated immune response after SCI.

Quercetin, an important flavonoid component of various herbs, has long been used as antioxidant and anti-inflammatory agent in traditional Chinese medicine [12–14]. It was confirmed to have neuroprotective effects on SCI by reducing death of neurons, decreasing neural tissue damage, and inhibiting inflammatory responses [15–18]. However, the effects of quercetin on survival of OLs and polarization of macrophages/microglia after SCI still remain unclear.

In this study, we investigated whether quercetin promoted OLs survival and suppressed the detrimental M1-type immune response after SCI, and explored the possible mechanism. We first showed that quercetin improved functional recovery after SCI. We then demonstrated that quercetin significantly reduced necroptosis of OLs after SCI without affecting apoptosis and regeneration of OLs. Meanwhile, quercetin significantly reduced myelin and axon loss after SCI. We further revealed that quercetin suppressed macrophages/microglia polarized to M1 phenotype through inhibition of STAT1 and NF-κB pathway, which contributes to the ameliorated necroptosis of OLs. Taken together, our study demonstrated that quercetin alleviated necroptosis of OLs in part by suppressing M1 macrophages/microglia polarization through inhibition of STAT1 and NF-κB pathway after SCI.

## Methods

### Experimental design

Male Sprague-Dawley rats (200–250 g) were purchased from the Laboratory Animal Center of Xi’an Jiaotong University. This study was carried out in accordance with the recommendations of ‘Animal Care and Use, Committee of Xi’an Jiaotong University.’ The protocol was approved by the ‘Committee of Xi’an Jiaotong University.’ A total of 120 rats were equally randomized into three groups: Sham group, SCI + saline group, and SCI + quercetin group. The sham group received only laminectomy, and the vehicle (1 ml sterile saline plus 1% DMSO) was administrated via intraperitoneal injection (i.p.) after SCI as SCI + saline group. According to the previous finding that functional recovery correlates treatment duration of quercetin after SCI [19], 7.5 mg/kg quercetin (Sigma-Aldrich, St. Louis, MO, USA) in 1 ml vehicle was administered i.p. twice daily for 10 days after SCI.

### Animal model of spinal cord injury

Rats were anesthetized with 1% sodium pentobarbital (60 mg/kg), and bilateral laminectomy of vertebrae T8 was performed to expose the spinal cord. The spinal cord crush model was made by a mechanical device [20]. Briefly, we used the forceps (53327T, 66 Vision-Tech Co., Ltd., China) mounted onto the device to clamp the cord, with the forceps tips of 0.5-mm width when fully closed, and the duration of the injury remained for 20 s. After crush, manual bladder expression was performed once a day, until reestablishment of micturition reflex. Depending on the following experiments, rats were sacrificed at 10 and 21 days post injury (dpi).

### Behavioral tests

#### Basso-Beattie-Bresnahan scores

The Basso-Beattie-Bresnahan (BBB) scores were used to test rat open-field locomotion at 1 day before, and 1, 3, 7, 10, 14, and 21 days after spinal cord injury (SCI) following methodology of BBB [21]. Functional scores ranges from 0 to 21 points in terms of the ankle, hip, knee, and trunk movement, as well as the coordination of each rat. The locomotion scores was observed and recorded by two independent observers blinded to the experiments.

#### Rump-height Index assay

The rats were video-recorded during walking through the left to right side on a runway bar (160 cm long, 10 cm wide, and 8 cm thick) at 1 day prior to and 1, 3, 7, 10, 14, and 21 days post-SCI. The rump-height Index (RHI) is defined as the height of the rump, normalized to the thickness of the beam measured along the same vertical line [22]. To minimize the variations of pre-surgery RHI of each animal, the standardized RHI (dividing post-injury value by pre-injury value) was applied for comparisons.

### Tissue processing

Seven days after SCI or at the end of the 21-day observation period, rats were anesthetized and trans-cardially perfused with 200 ml of saline, followed by 400 ml of 4% PFA. Subsequently, 2-cm spinal cord segments encompassing the lesion site were obtained and cryoprotected in sucrose (30% in 0.1 M phosphate buffer, pH 7.4) until the blocks sank.

Both serial transverse sections (12-μm-thick) and serial sagittal sections (12-μm-thick) were cut on a cryostat and prepared for immunostaining.

### Immunohistochemistry

After blocking in 0.01 M PBS containing 3% BSA and 0.3% Triton X-100 for 30 min, sections were incubated with primary antibodies in humidified boxes overnight at 4 °C. The primary antibodies used in the experiment included rabbit anti-RIP3 (1:500, ENZO, New York, NY, USA), rat anti-MLKL (1:200, Millipore, Billerica, MA, USA), rabbit anti-phosphorylated MLKL (pMLKL) (1:300, Abcam, Cambridge, Cambridgeshire, UK), mouse anti-CC1 (1:600, Milipore, Billerica, MA, USA), rabbit anti-iNOS (1:200, Abcam, Cambridge, Cambridgeshire, UK), goat anti-Arginase1(1:200, Santa Cruz Biotechnology, Dallas, Texas, USA), mouse anti-glial fibrillary acidic protein (GFAP,1:2000, Sigma-Aldrich, St. Louis, MO, USA), rabbit anti-MBP(1:200, Abcam, Cambridge, Cambridgeshire, UK), mouse anti-NF200 (1:200,Abcam, Cambridge, Cambridgeshire, UK), rabbit anti-Cleaved Caspase-3(1:200, Cell Signaling, Danvers, MA, USA), goat anti-Iba-1(1:200,Abcam, Cambridge, Cambridgeshire, UK), mouse anti-PDGFRα (1:200, Cell Signaling, Danvers, MA, USA), rat anti-BrdU (1:300,Abcam, Cambridge, Cambridgeshire, UK), and rabbit anti-Olig2 (1:200, Abcam, Cambridge, Cambridgeshire, UK). The sections were then incubated with the corresponding secondary antibodies conjugated with Alexa Fluor 594 (donkey anti-rabbit IgG or donkey anti-mouse IgG, 1:1000, Jackson ImmunoResearch, West Grove, PA, USA), Alexa Fluor 488 (donkey anti-rat IgG or donkey anti-mouse IgG, 1:1000, Jackson mmunoResearch, West Grove, PA, USA) in a dark environment for at RT 1 h. The sections were counterstained with Hoechst 33342 to identify all nuclei. Sections were examined and photographed under a confocal microscope (LSM 800, Zeiss, Oberkochen, Germany).

### BrdU incorporation and detection

For detection of regeneration of OLs, BrdU (100 mg/kg, Sigma, St. Louis, MO, USA) injection was initiated at 24 h after injury, once a day for 9 days. Rats were sacrificed at 10 days after injury. HCl treatment (2 N HCl, 30 min at 37 °C) was performed for BrdU staining.

### Luxol fast blue staining

For Luxol fast blue (LFB) staining, serial transverse cryosections (12 μm thickness) were stained as previously described. Briefly, slides were incubated in 0.1% LFB (Sigma, St. Louis, MO, USA) in acidified 95% ethanol overnight at 60 °C. The slides were then differentiated and counterstained with 0.05% lithium carbonate and cresyl violet solution. LFB-stained sections at 2000 μm rostral and caudal to the injury site, as well as at the lesion epicenter, were analyzed for myelin sparing. The spared myelin in white matter was quantified by Image pro-plus (Media Cybernetics, Silver Spring, USA).

### Electron microscopic study

Rats were deeply anesthetized with 1% sodium pentobarbital intraperitoneally (60 mg/kg body weight) and perfused with 200 ml saline, followed by 500 ml 4% PFA containing 0.05% glutaraldehyde. Then the injured cord was removed and postfixed in the same fixative solution for 4 h at 4 °C. Serial coronal sections of 50 μm thickness were prepared with a vibratome (VT 1000S, Leica, Wetzlar, Hesse, Germany), then placed in 0.01 M PBS containing 25% sucrose and 10% glycerol for 2 h for cryoprotection. The sections were then immersed in 0.5% osmium tetroxide for 1 h, dehydrated with graded ethanol series, then in propylene oxide, and finally flat-embedded in Epon812 (SPI-CHEM). After trimming under a stereomicroscope, the sections were glued onto blank resin stubs. Serial ultrathin sections were cut with an ultramicrotome (Leica EM UC6, Leica, Wetzlar, Hesse, Germany) and mounted on mesh grids. They were then counterstained with uranyl acetate and lead citrate, observed, and photographed under an electron microscope (JEM-1230, JEOL Ltd., Akishima, Tokyo, Japan) equipped with CCD camera and its application software (832 SC1000, Gatan, Pleasanton, CA, USA).

### Axonal diameter and G ratio determination

Axonal diameter was determined from counting of about 200 axons in white matter of the injured segments of three rats at 21 dpi using Image J software. G-ratio was determined as the ratio of inner axonal diameter to the total outer diameter of each axon fiber of about the same 200 axons of three rats at 21 dpi.

### Primary microglial culture and polarization

Microglia were isolated from P2 Sprague-Dawley rat pups as described. Briefly, the spinal cord was dissected, mechanically dissociated, and then digested with 0.125% trypsin/0.02% EDTA. Cells were plated onto poly-d-lysine-coated culture flasks and cultured in DMEM (Gibco, Waltham, USA) containing 10% FCS at 37 °C with 5% CO_2_. The medium was exchanged every 3 days and at about 8th day when cells became confluent, the flasks were shaken with a shaker at 260 rpm for 0.5 h to obtain microglia. The quality of microglia was analyzed by immunostaining of Iba1 (> 90% Iba1^+^).

After about 7 days culture, microglial cells were treated with LPS (100 ng/ml, Sigma, Santa Clara, USA) and IFN-γ (20 ng/ml, Peprotech, Rocky Hill, USA) for M1 polarization, or quercetin plus LPS and IFN-γ, followed by medium replacement. Twenty-four hours later, the conditioned medium (M1 CM and Q-M1 CM) was obtained.

### Primary oligodendrocytes culture

After microglia was harvested, with another overnight shaking at 280 rpm, cell suspension was harvested and seeded on tissue culture dishes (Corning, NY, USA) for 40 min. The dishes were then gently swirled every 6 min to remove contaminating astrocytes and microglia. The non-adherent cells were seeded onto poly-d-lysine-coated culture dishes in DMEM containing 10% FCS at density of 5 × 10^5^ cells/cm^2^ for 3 h, and the medium was then changed by oligodendrocyte precursor cells (OPCs) proliferation medium (chemically defined medium with 10 ng/ml PDGF-AA and 10 ng/ml bFGF). Three days later, OPCs were isolated with DMEM/F12 containing 0.01% EDTA, 0.2 mg/ml DNase I, and 5 mg/ml insulin, and seeded in differentiation medium (chemically defined medium with 30 nM triiodothyronine and 5 mg/ml *N*-acetyl-l-cysteine). Mature oligodendrocytes (OLs) were obtained 5 days later. Chemically defined medium contains high glucose DMEM/F12 supplemented with 4 mM l-glutamine, 50 mg/ml transferrin, 1 mM sodium pyruvate, 5 mg/ml insulin, and 10 ng/ml d-biotin. The mature OLs were identified by MBP staining.

### Western blotting

About 1.5 cm length of injured cord segments or primary cultured microglia were harvested and homogenized with ice-cold radio-immunoprecipitation assay (RIPA) lysis buffer. Proteins were separated in 10% SDS–PAGE gel, and transferred onto polyvinylidene difluoride (PVDF) membrane. The membranes were first blocked with 5% non-fat milk in TBST for 1 h at RT, and incubated with primary antibodies for RIP3 (1:800, ENZO, New York, NY, USA), MLKL (1:500, Millipore, Billerica, MA, USA), pMLKL (1:500, Abcam, Cambridge, Cambridgeshire, UK), iNOS(1:500, Abcam, Cambridge, Cambridgeshire, UK), p-STAT1 (1:1000, Cell Signaling, Danvers, MA, USA), nuclear factor-kappa B (NF-κB) (P65,1:1000, Cell Signaling, Danvers, MA, USA), phospho-NF-κB p65/p-P65 (1:1000, Cell Signaling, Danvers, MA, USA), or beta-actin (1:5000, Sigma, Santa Clara, USA) at 4 °C overnight. The membranes were then incubated with corresponding horseradish peroxidase-conjugated secondary antibodies (1:5000, Jackson ImmunoResearch, West Grove, PA, USA) at RT for 1 h. The immunoreactive bands were scanned with the Bio-Rad Image Lab system. The expression level was measured by ImageJ software (NIH, USA) corresponding to the band intensities.

### Quantitative reverse transcription polymerase chain reaction

Total RNA was extracted from 1.5 cm length of injured cord segments or microglia using TRizol (Invitrogen, Carlsbad, CA, USA) following the manufacturer’s instructions. Complementary DNA synthesis was performed using a Reverse Transcriptase kit (Invitrogen, Carlsbad, CA, USA). Real-time PCR for TNFα, iNOS, CD86, CD206, Arginase1, IL-4, IL-10, IL-12, IL-1β, and TGFβ were performed with SYBR Green (TaKaRa, Dalian, China) using Bio-Rad 96 Real-time PCR system (Bio-Rad Laboratories, Hercules, CA, USA), with GAPDH used as a reference control. The primer sequences are listed in Table 1.

**Table 1:** PCR primer sequences

Gene	Forward primer(5′-3′)	Reverse primer (5′-3′)
TNFα	ATGGGCTCCCTCTCATCAGT	GCTTGGTGGTTTGCTACGAC
iNOS	TCCTCAGGCTTGGGTCTTGT	ATCCTGTGTTGTTGGGCTGG
CD86	AGACATGTGTAACCTGCACCAT	TACGAGCTCACTCGGGCTTA
CD206	TCAACTCTTGGACTCACGGC	CATGATCTGCGACTCCGACA
Arg1	CCAGTATTCACCCCGGCTAC	GTCCTGAAAGTAGCCCTGTCT
IL-4	GTACCGGGAACGGTATCCAC	TGGTGTTCCTTGTTGCCGTA
IL-10	CGACGCTGTCATCGATTTCTC	CAGTAGATGCCGGGTGGTTC
IL-12	ATCATCAAACCGGACCCACC	AGGAACGCACCTTTCTGGTT
IL-1β	GCTTCCTTGTGCAAGTGTCT	TCTGGACAGCCCAAGTCAAG
TGFβ	CTGCTGACCCCCACTGATAC	AGCCCTGTATTCCGTCTCCT
GAPDH	AGTGCCAGCCTCGTCTCATA	GGTAACCAGGCGTCCGATAC

### Propidium iodide staining

After the cells were treated with M1 CM and Q-M1 CM, propidium iodide (PI) (5 μg/ml, Sigma, Santa Clara, USA) and Hoechst 33342 (3 μg/ml, Sigma, Santa Clara, USA) were added into the culture medium and incubated for 20 min at 37 °C. After washing with 0.01 M PBS three times, cells were fixed with 4% PFA for 10 min at RT. Cells were then imaged and counted under inverted fluorescence microscope (IX71, Olympus, Tokyo, Japan).

### ROS measurement

DCFH-DA probe (Sigma, St. Louis, MO, USA) was used to detect reactive oxygen species (ROS) levels. DCFH-DA (10 μM) was added to 96-well plates seeded with OLs incubated for 30 min at 37 °C. ROS levels were then examined by a multimode microplate reader (TECAN, Infinite M200, Mannedorf, Switzerland).

### ATP measurement

ATP levels were measured using the Cell Titer-Glo assay kit (Promega, Madison, USA). Equivalent Cell Titer-Glo Reagent was added into medium for 5 min to induce cell lysis, followed by incubating for 15 min to stabilize luminescent signal. Luminescence was detected by multimode microplate reader (TECAN, infinite M200, Mannedorf, Switzerland).

### Quantification of cells

In our study, the method of profile counts followed by calibration with the empirical method was adopted for unbiased data [23, 24].The 12-μm thickness of the section ensures that the plane of central canal is cut through on every slide, making random selection of the slide possible. For cells quantification, all of the positive cells in the defined area in all sections of the randomly chosen slide were counted. Then calibration of the profile numbers was made by empirical method. Cell counting was performed by an observer who was blinded to the experiment design.

For quantitative analysis of density of MBP and NF200, serial transverse cryosections (12-μm thickness) were collected every millimeter section rostral and caudal 2000 μm to the lesion site. The longitudinal sections were also performed to confirm the density of MBP. The data were presented as a percentage relative to that in sham control (100%).

### Statistics

Quantification was performed by researchers who were blinded to the group allocation. No data were excluded from the analysis, except for five rats evaluated by BBB scores. In the present study, data are expressed as the mean ± standard error of the mean (SEM). Comparisons between two groups were evaluated by the Student’s *t* test [25]. For multiple comparisons with the three or four groups, we performed one-way analysis of variance (ANOVA), followed by Tukey post-hoc tests, except for BBB scoring and RHI assay which were further analyzed by the Bonferroni post hoc test as recommended [26, 27]. Differences were considered statistically significant if *p < 0.05, **p < 0.01, or ***p < 0.001.

## Results

### Quercetin improved functional recovery after SCI

**Fig. 1:**
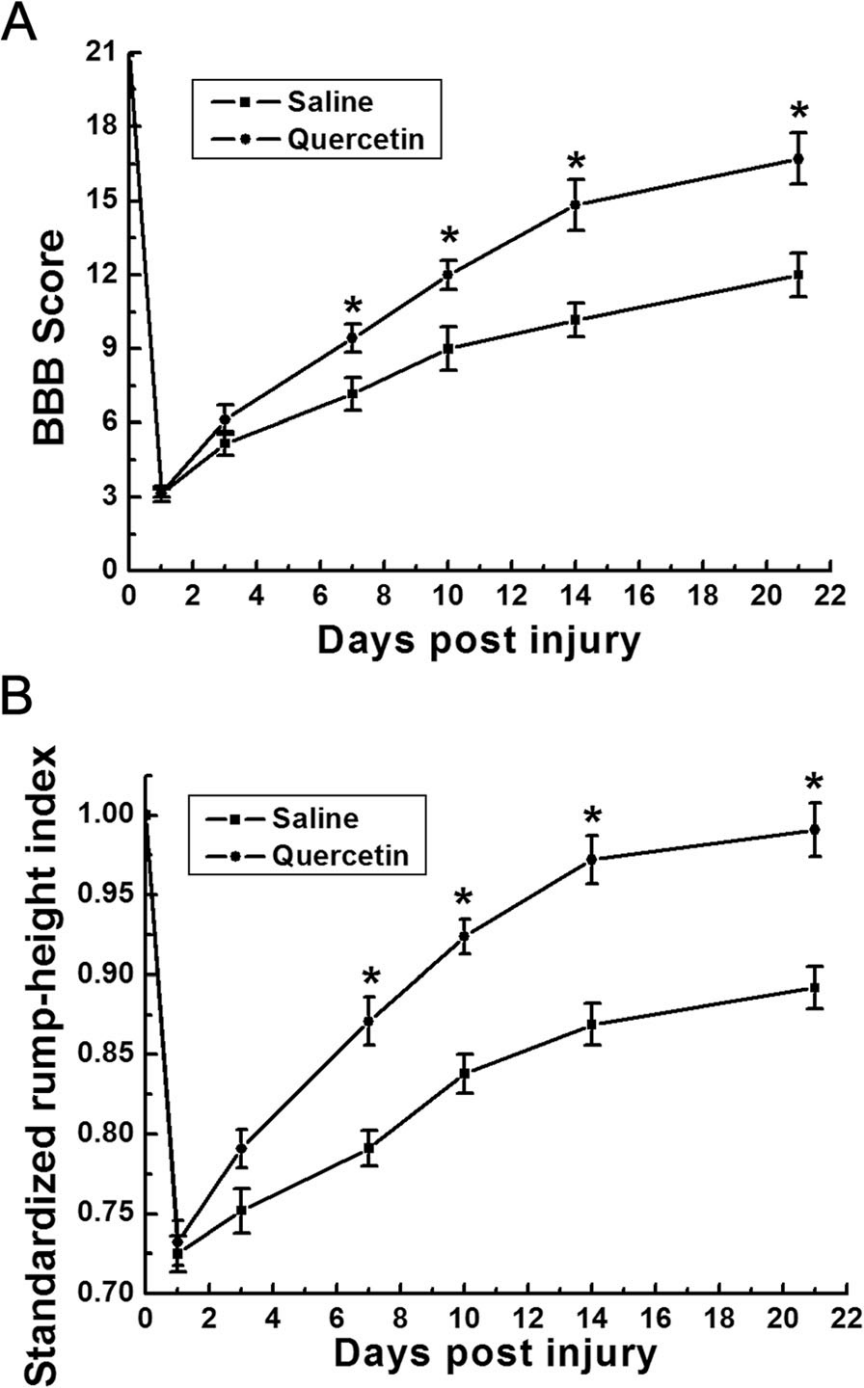
Quercetin improved the locomotion recovery after SCI. **a** BBB scores at 1, 3, 7, 10, 14, and 21 days post SCI. The BBB scores in quercetin-treated group were significantly higher than those in control group at 7, 10, 14, and 21 days after injury. **b** SRHI values at 1, 3, 7, 10, 14, and 21 days post SCI. The rump height was significantly elevated in quercetin-treated rats at 7, 10, 14, and 21 days after injury. All data are expressed as mean ± SEM. Differences among groups were determined with one-way ANOVA followed by the Bonferroni post hoc test. *N* = 6 in each group, *p < 0.05

Locomotor functional recovery was evaluated using BBB scoring and RHI assay at 1 day before injury as well as 1, 3, 7, 10, 14, and 21 days after SCI. Compared with SCI + saline control, the quercetin-treated rats showed significant higher BBB scores from 7 dpi (*n* = 6, *p <0.05, Fig. 1a), which was consistent with the previous results [16]. Similarly, the rump height shown by RHI was significantly elevated from 7 dpi within quercetin-treated group (*n* = 6 rats/group, *p < 0.05, Fig. 1b).

### Quercetin prevented necroptosis of OLs without affecting apoptosis and regeneration of OLs after SCI

**Fig. 2:**
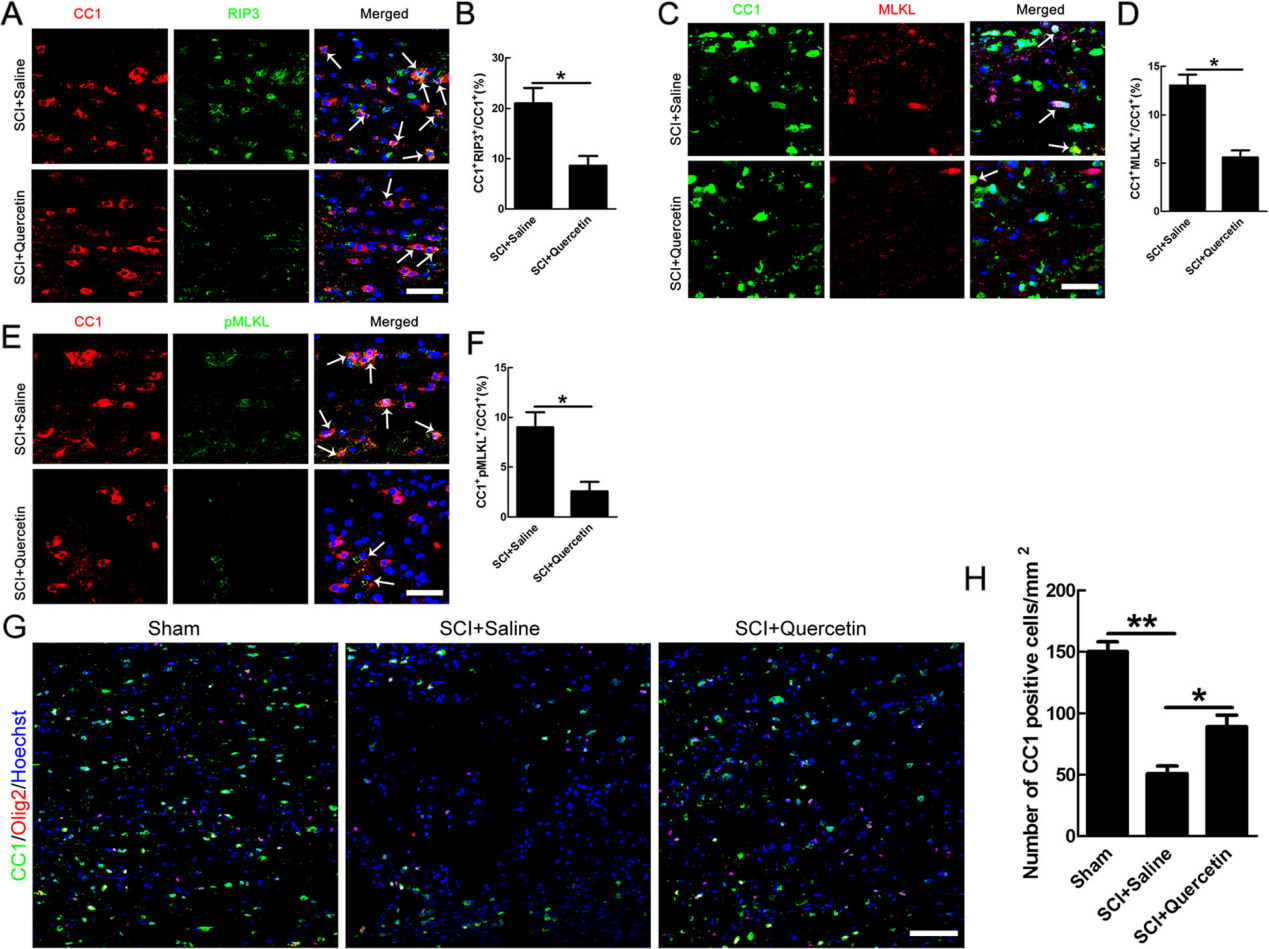
Quercetin inhibited necropotosis of OLs after SCI. **a**, **c**, **e** Double-immunostaining of CC1 with RIP3, MLKL, or pMLKL in saline or quercetin-treated rats at 10 dpi. Representative images are from sections 100 μm to the lesion epicenter. Arrows indicate double-positive cells. Scale bar = 30 μm. **b**, **d**, **f** Quantification of CC1/RIP3-, CC1/MLKL-, and CC1/pMLKL-positive cells. Notice the decrease of double-positive cells in quercetin-treated rats. *p < 0.05 compared to SCI + saline control. **g**, **h** Immunostaining and quantification of CC1-positive cells in sham, saline, or quercetin-treated rats at 10 dpi. Scale bar = 100 μm. Notice the increase of CC1-positive cells in quercetin-treated rats, compared to saline-treated rats. All data are expressed as mean ± SEM. Differences among groups were determined with unpaired two-tailed *t* test (**b**, **d**, **f**) or one-way ANOVA followed by Tukey post-hoc test (**h**). *N* = 6 in each group, *p < 0.05, ** *p*<0.01

Given that the survival of OLs is crucial for functional recovery after SCI [4, 28], we then focused on death and regeneration of OLs. Apart from apoptosis, RIP3/MLKL-mediated necroptosis is activated after SCI and our previous study showed that CC1/MLKL double-positive cells appeared after SCI [9], indicating the necroptosis of OLs. To investigate the effect of quercetin on necroptosis of OLs after SCI, double staining of RIP3/CC1, MLKL/CC1, and pMLKL/CC1 were performed at 10 days post injury. The data of double staining showed that the number of RIP3/CC1, MLKL/CC1, and pMLKL/CC1 was reduced by 59.05 ± 3.57%, 56.92 ± 3.59%, 43.3 ± 1.07%, and 71.11 ± 3.82% respectively after quercetin treatment (*n* = 6, *p < 0.05, Fig. 2a–f). Moreover, quercetin improved OLs survival by about 80% shown by CC1-positive cells in our model (*n* = 6, *p < 0.05, Fig. 2g, h). None of the RIP3-, MLKL-, and pMLKL-positive cells were observed in sham-operated cords (data not shown). The above results indicated that quercetin treatment significantly reduced the numbers of necroptosis of OLs after SCI.

**Fig. 3:**
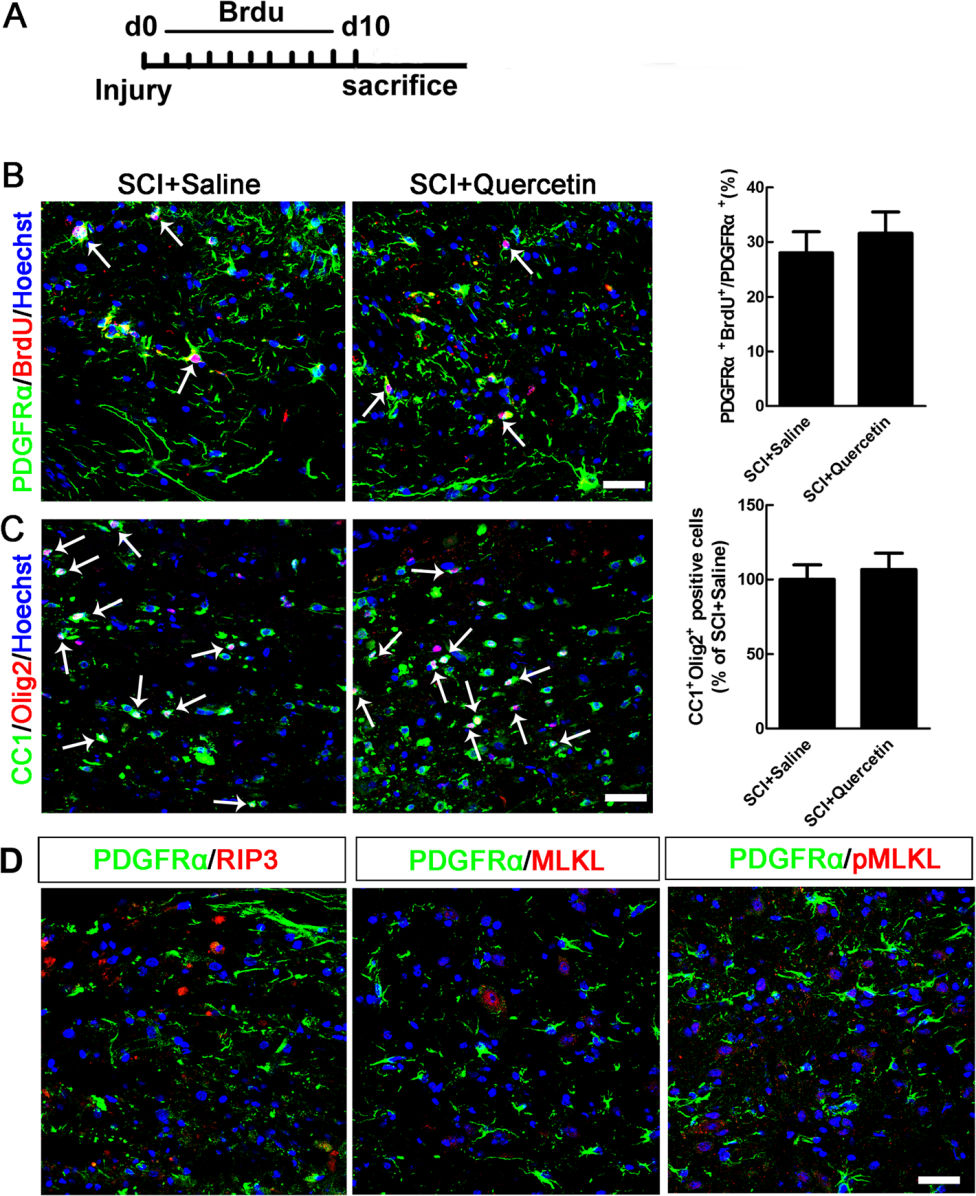
Quercetin had no effect on regeneration and apoptosis of OLs after SCI. **a** BrdU administration scheme. **b**, **c** Co-immunostaining of PDGFRα/BrdU and CC1/Olig2, and quantification of double-positive cells. Note that no significant effect of quercetin on OL regeneration after SCI. **d** Double-immunostaining of PDGFRα and RIP3, MLKL, or pMLKL. Notice that none of the double-positive cells was found. Scale bars = 50 μm. All data are expressed as mean ± SEM. Differences among groups were determined with unpaired two-tailed *t* test (**b**, **c**). *N* = 6 in each group

Further, we examined that whether quercetin could improve OL preservation only or enhance both OL preservation and OL regeneration after SCI. BrdU administration scheme was shown in Fig. 3a. By co-immunostaining of PDGFRα/BrdU and CC1/Olig2, we found that quercetin had no significant effect on OL regeneration after SCI (Fig. 3b, c). In addition, by co-immunostaining of PDGFRα and RIP3, MLKL, or pMLKL, we found that there are none of the double-positive cells (Fig. 3d), indicating that none of the OPCs occurred necroptosis.

Moreover, we showed that just a small proportion of OLs(about 2.3%) died through apoptosis in the first 5 days after SCI, and from 5 dpi to 10 dpi, more OLs were eliminated by necroptosis (about 13%) rather than apoptosis (about 3.1%) (Additional file 1: Figure S1a–c), indicating that necroptosis was the main way of OLs loss in our model of SCI. In addition, no significant effect of quercetin on apoptosis of OLs was found (Additional file 1: Figure S1d-e).

### Quercetin reduced myelin and axonal loss after SCI

**Fig. 4:**
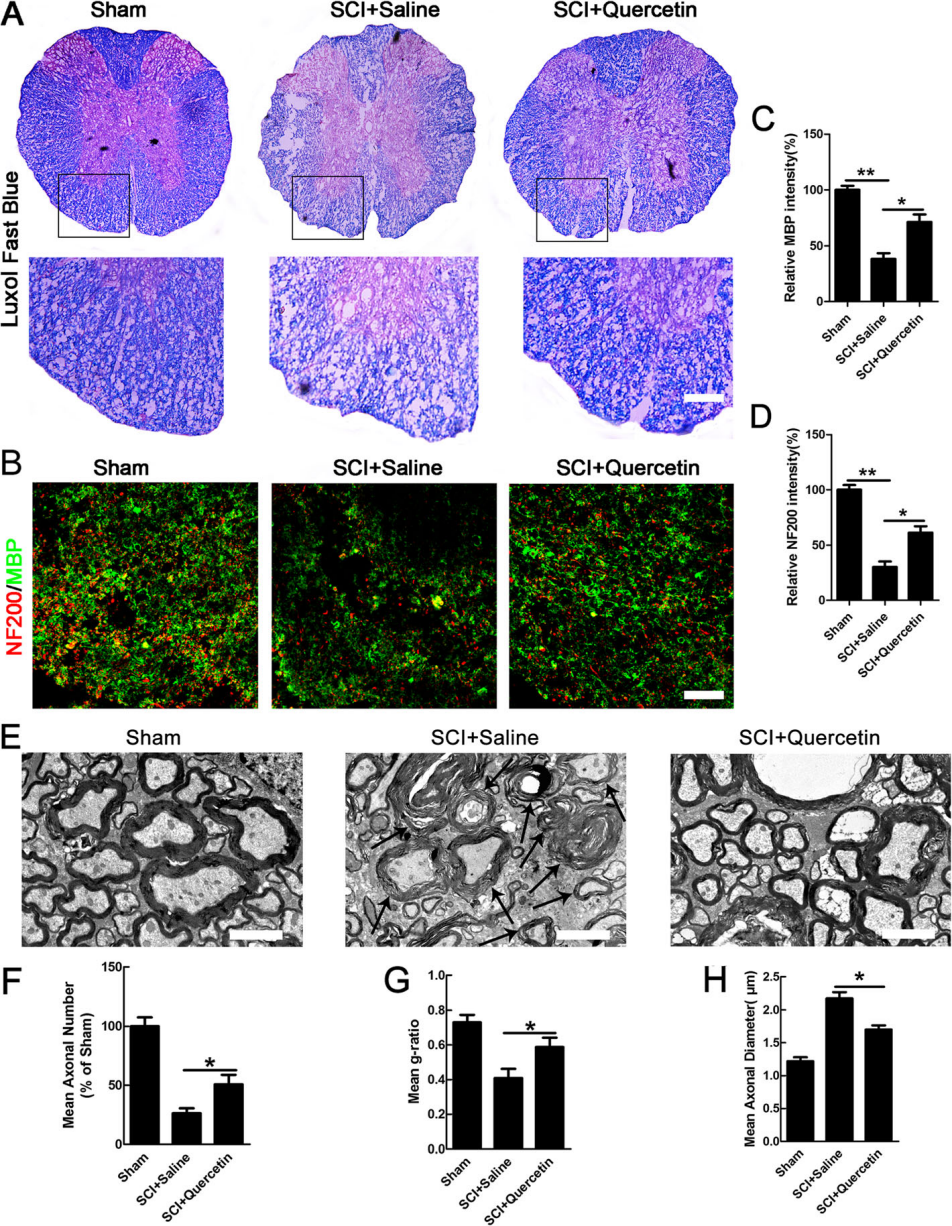
Quercetin reduced myelin and axonal loss after SCI. **a** Spinal cords at 21 dpi were processed for Luxol fast blue. **b** Double-staining of MBP/NF200 in transverse sections. Immunofluorescence image of MBP and NF200 in lateral and dorsal funiculus of cords in sham, saline, or quercetin-treated rats at 21 dpi after SCI. Transverse cryosections were selected 2000 μm rostral to the lesion site. Scale bar = 100  μm. **c**, **d** Quantification of MBP and NF200 intensity. Note that quercetin treatment attenuated the reduction of MBP and NF200 in the white matter after injury. **e**–**h** Electron microscopic images and quantification of the mean axonal numbers, mean g-ratios, and mean axonal diameters in spinal cord white matter at 21 dpi. Arrows indicate degenerated myelin exhibiting onion-like appearance whose myelin lamellae were disorganized and loosened. Note that the increased number of axonal numbers, mean g-ratios, and decreased mean axonal diameters in quercetin treated rats. Scale bar = 2 μm. All data are expressed as mean ± SEM. Differences among groups were determined with one-way ANOVA followed by Tukey post-hoc test (**c**, **d**, **f**, **h**). *N* = 6 in each group, *p < 0.05; **p < 0.01

Given that death of OLs results in demyelination and secondary axon damage [29, 30], we examined the effect of quercetin on loss of myelin and axons after SCI. Spinal cords at 21 days after injury were processed for LFB staining and immunostaining to assess the myelin loss(Fig. 4a, b). In both dorsal and lateral funiculus of the white matter at 2000 μm rostral from the lesion and longitudinal sections, myelin and axonal loss was extensive in the SCI + saline control, whereas quercetin treatment significantly attenuated MBP-positive myelin and NF200-positive axonal loss (*n* = 6; **p < 0.01, *p < 0.05, Fig. 4a–d). Demyelination was also observed in the same region of white matter by electron microscopic analysis, and the data showed that SCI induced decompaction of myelin sheaths with a decreased g-ratio, an increased large-diameter axons, and a decreased axonal number, whereas quercetin treatment attenuated these effects(*n* = 6; *p < 0.05, Fig. 4e–h). These data indicated that quercetin treatment markedly reduced the extent of both myelin and axon loss after SCI.

### Quercetin influenced the macrophages/microglia M1-M2 polarization balance after SCI

**Fig. 5:**
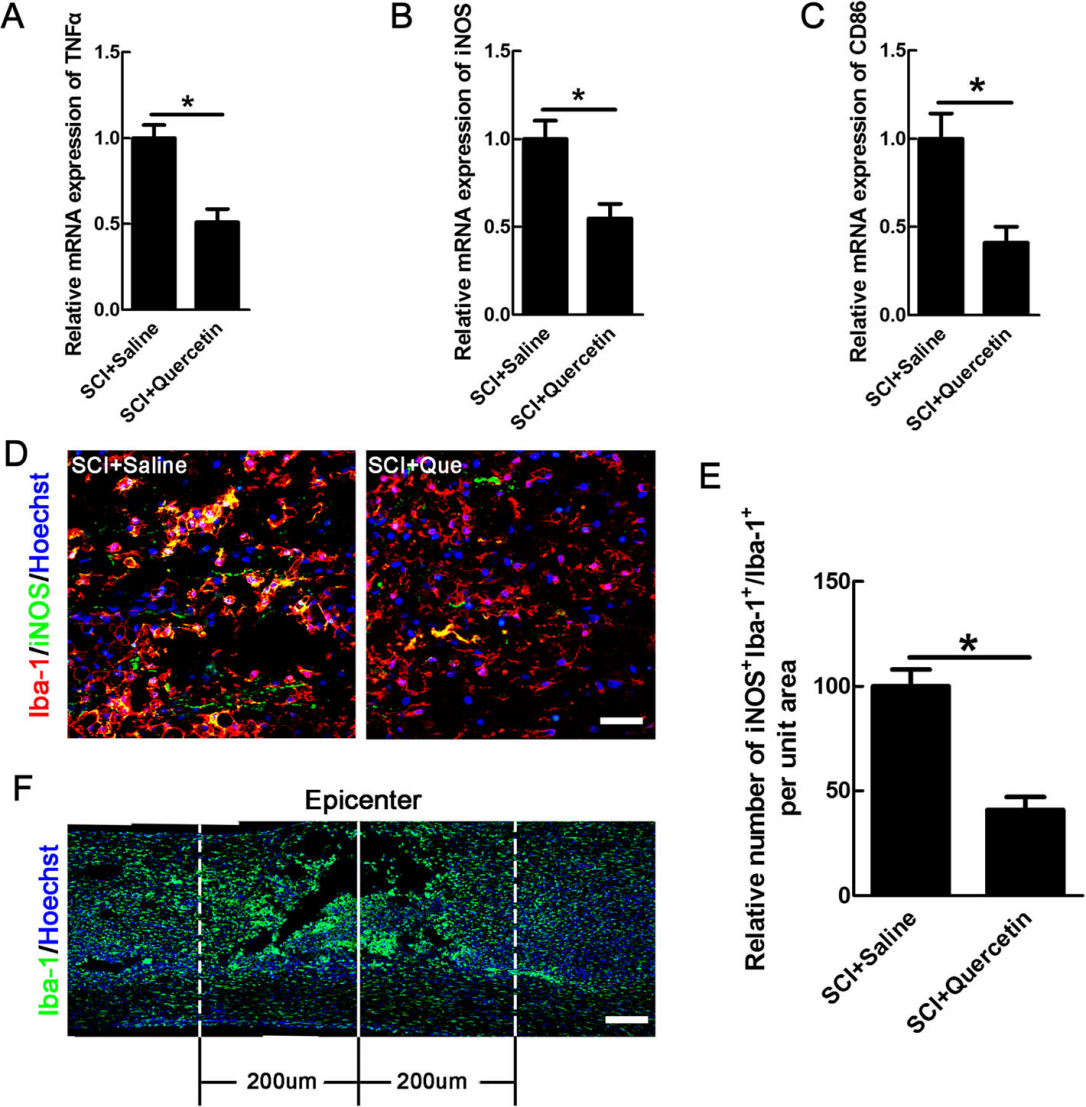
Quercetin reduced the numbers of M1-type macrophages/microglia in injured spinal cord. **a**–**c** Real-time reverse-transcriptase polymerase chain reaction of M1-associated mRNA transcripts of TNFα, iNOS, and CD86 in saline or quercetin-treated rats at 10 dpi. **d** Double-staining of iNOS and Iba-1 in quercetin-treated or saline-treated rats at 10 dpi. Representative images are from sections 20 μm to the lesion epicenter. Scale bar = 30 μm. **e** Quantification of iNOS-positive microglia/macrophages. Notice the decrease of iNOS-positive microglia/macrophages in quercetin-treated rats. *p < 0.05 compared to SCI + saline control. **f** The bilateral areas 200 μm rostral and caudal to the lesion site and the lesion epicenter on which quantification was performed. Scale bar = 80 μm. All data are expressed as mean ± SEM. Differences among groups were determined with unpaired two-tailed *t* test (**a**–**c**, **e**). *N* = 6 in each group, *p < 0.05

Considering that the ameliorated cell necroptosis needs a permissive immuno-microenvironment and quercetin has been used as an anti-inflammatory agent [10, 31], we then explored the effects of quercetin on macrophages/microglia-mediated inflammation after SCI. The activation of macrophages/microglia can be divided to pro-inflammatory, damaging M1 phenotype, and anti-inflammatory, regeneration-promoting M2 phenotype after SCI [32]. Because the anti-inflammatory effect of quercetin has been reported in SCI [17, 33], we speculated that quercetin could inhibit macrophages/microglia polarization to M1 phenotype. To determine the effect of quercetin on M1 polarization, we first examined the mRNA levels of TNFα, iNOS, and CD86, the markers of M1 macrophages/microglia at 10 days after injury. Our data showed that mRNA of TNFα, iNOS, and CD86 was decreased by 50.17 ± 2.43%, 45.23 ± 2.06%, and 59.14 ± 3.51% respectively in the quercetin group (*n* = 6 rats/group, *p < 0.05, Fig. 5a–c). Meanwhile, quantification in bilateral areas 200 μm rostral and dorsal to lesion center, as shown in Fig. 5f, demonstrated that quercetin decreased the number of iNOS-expressing macrophages/microglia by 59.1 ± 2.26% (*p < 0.05, Fig. 5d, e).

**Fig. 6:**
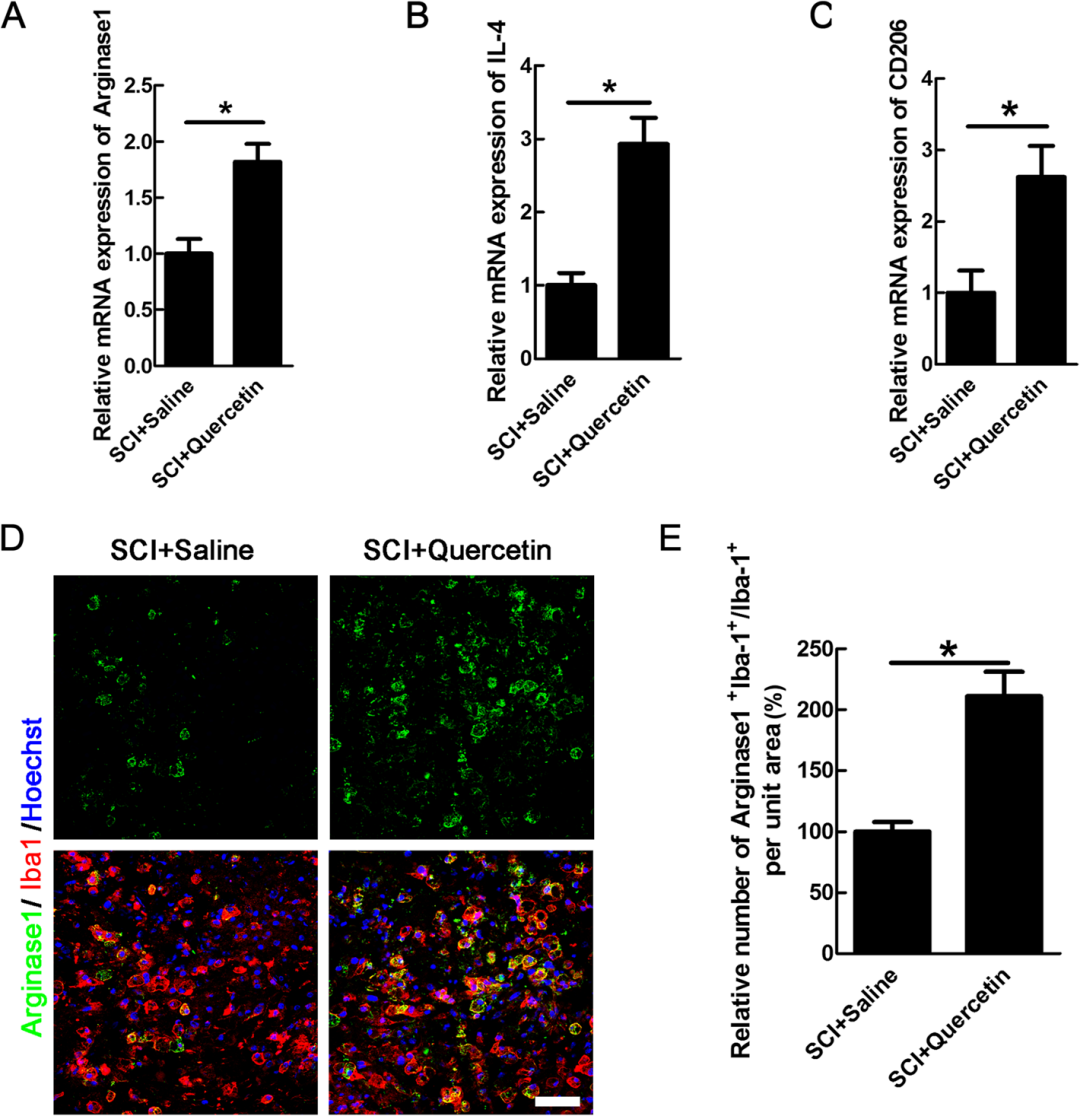
Quercetin increased the numbers of M2-type macrophages/microglia after SCI. **a**–**c** Expression of M2-associated mRNA transcripts of Arginase1, IL-4, CD206 in saline, or quercetin-treated rats at 10 dpi. **d** Double-staining of Arginase1 and Iba-1 in quercetin-treated or saline-treated rats at 10 dpi. Scale bar = 30 μm. **e** Quantification of the numbers of Arginase1-positive microglia/macrophages. Sections were taken from the lesion epicenter. All data are expressed as mean ± SEM. Differences among groups were determined with unpaired two-tailed *t* test (**a**–**c**, **e**). *N* = 6 in each group, *p < 0.05

We further examined the effect of quercetin on M2 polarization. Interestingly, the results showed that quercetin increased the mRNA levels of Arginase1, IL-4, and CD206 by 45.09 ± 2.14%, 65.89 ± 5.34%, and 61.9 ± 3.75% respectively (*n* = 6 rats/group, *p < 0.05, Fig. 6a–c). Because M2 macrophages/microglia was mainly located in the epicenter after SCI [34, 35], we analyzed the numbers of M2 in the inner border of GFAP-immunoreactive zone (Additional file 2). The result showed that the Arginase1-positive macrophages/microglia was increased by 46.44 ± 4.79% (*p < 0.05, Fig. 6d, e). None of the iNOS- or Arginase1-positive cells was observed in sham-operated cords (data not shown). Collectively, the above results showed that quercetin could inhibit macrophages/microglia polarization to M1 phenotype, while promote M2 polarization, which may forms an environment allowing for survival of OLs.

### The mechanism of effect of quercetin on M1 macrophages/microglia-induced necroptosis of OLs

**Fig. 7:**
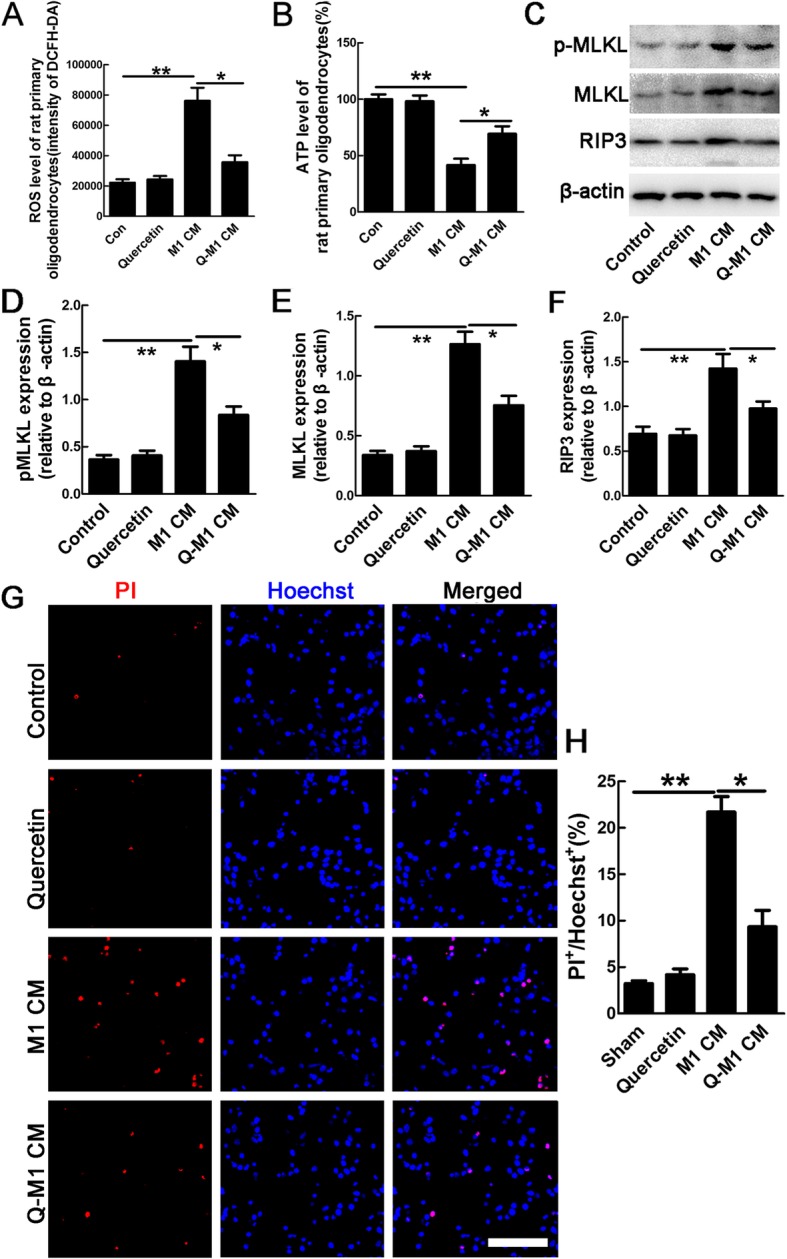
Quercetin inhibited M1 macrophages/microglia induced-necroptosis of OLs. **a**, **b** Quantification of intracellular ROS and ATP levels in OLs, or cells treated by quercetin alone, conditioned medium (CM) from M1 microglia (M1 CM) and quercetin-treated M1 microglia (Q-M1 CM). Notice that quercetin significantly attenuated the effects of M1 CM on necroptosis of OLs. **c**–**f** Quantification of the expression levels of RIP3, MLKL, and pMLKL in control cells, quercetin alone-treated, M1 CM-treated, or Q-M1 CM-treated cells. β-actin was used as a loading control. Notice that quercetin significantly decreased the expression of necroptotic markers induced by M1 CM. **g**, **h** PI staining and quantification of PI-positive cells in control cells, or cells treated by quercetin alone, M1 CM, and Q-M1 CM. Notice that quercetin significantly decreased the number of PI-labeled cells induced by M1 CM. Scale bar = 50 μm. Results are mean ± SEM of three independent experiments. Differences among groups were determined with one-way ANOVA followed by Tukey post-hoc test (**a**, **b**, **d**–**f**, **h**). *p < 0.05; **p < 0.01

The pro-inflammatory response is always accompanied with cell death after SCI [36]. To explore the direct evidence that M1 macrophages/microglia contributes to necroptosis of OLs, we further cultured OLs and microglia and induced necroptosis. MBP staining was performed to identify the purity of OLs, and about 80% of the cells are MBP positive (data not shown). Quercetin-modified M1 CM (Q-M1 CM) was obtained by addition of quercetin to microglia when polarizing them to M1 phenotype. After treatment with conditioned medium of M1 microglia (M1 CM), the ROS level of OLs increased 3.3-fold, ATP level decreased 2.4-fold, PI labeled cells increased 6.7-fold, which were the indicators of necroptosis [37, 38] (*n* = 3, **p < 0.01, Fig. 7a, b, g, h). The expression of RIP3, MLKL, and p-MLKL was also significantly enhanced by M1 CM (*n* = 3, **p < 0.01, *p < 0.05, Fig. 7c–f). The effects were significantly blocked by Q-M1 CM, and quercetin alone has no significant effects on necroptosis of OLs (*n* = 3, *p < 0.05, Fig. 7a–h). These data suggested that quercetin inhibited necroptosis of OLs induced by M1 macrophages/microglia.

**Fig. 8:**
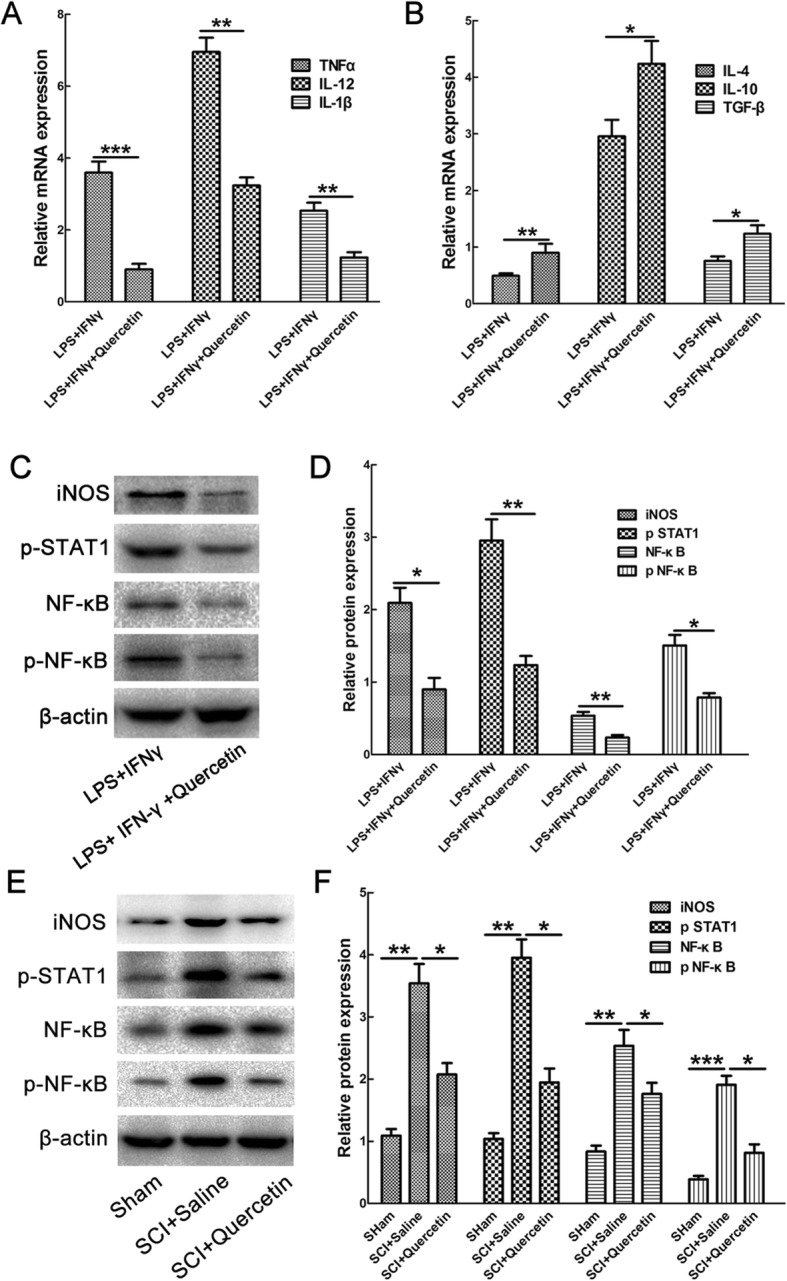
Effects of quercetin on M1 polarization of microglia and expression of pSTAT1, NF-κB, and pNF-κB. **a**, **b** The effect of quercetin on mRNA levels of M1 related TNFα, IL-12, IL-1β, and M2-related IL-4, IL-10, and TGF-β in M1 microglia. Note that quercetin significantly decreased mRNA levels of TNFα, IL-12, and IL-1β, while increased mRNA levels of IL-4, IL-10, and TGF-β. Data were expressed as mean ± SEM of three independent experiments. **c** Expression of iNOS, pSTAT1, NF-κB, and pNF-κB in microglia treated by LPS + IFN-γ in the presence or absence of quercetin. **d** Quantification of protein levels. β-actin was used as a loading control. Data were expressed as mean ± SEM of three independent experiments. **e** Expression of iNOS, pSTAT1, NF-κB, and pNF-κB in sham, saline, or quercetin-treated rats at 10 dpi after SCI. **f** Quantification of protein levels. Data were expressed as mean ± SEM of 6 rats. Differences among groups were determined with unpaired two-tailed *t* test (**a**, **b**, **d**) or one-way ANOVA followed by Tukey post-hoc test (**f**). *p < 0.05; **p < 0.01; ***p < 0.001

To determine whether this reduction resulted from the suppressed polarization of M1 macrophages/microglia, mRNA levels of M1-related markers and M2-related markers were detected. The results showed that quercetin decreased the mRNA levels of TNFα, IL-12, and IL-1β in M1 microglia, while increased mRNA levels of IL-4, IL-10, and TGF-β (*n* = 3, ***p < 0.001, **p < 0.01, *p < 0.05, Fig. 8a, b). The data provides the direct evidence that quercetin prevented macrophages/microglia polarized to M1 phenotype.

Further, we explored the possible mechanism of the above effect by examining the STAT1 and NF-κB signaling pathway, which was reported to be involved in transcriptional regulation of M1polarization of macrophages/microglia. In vitro, the expression of iNOS, pSTAT1, NF-κB, and p-NF-κB was decreased by 56.94 ± 2.49%, 58.31 ± 5.86%, 56.6 ± 3.46%, and 53.33 ± 9.58% respectively when quercetin was added to M1 microglia (*n* = 3, **p < 0.01, *p < 0.05, Fig. 8c, d). In vivo, SCI dramatically induced the expression of iNOS, pSTAT1, NF-κB, and p-NF-κB, and quercetin treatment significantly reduced their expressions by 42.86 ± 4.01%, 51.28 ± 2.41%, 30.43 ± 3.46%, and 57.37 ± 6.16% respectively (*n* = 3, **p < 0.01, *p < 0.05, Fig. 8e, f).

## Discussion

In this study, we demonstrated that quercetin treatment improved functional recovery after SCI. Quercetin also attenuated RIP3/MLKL-mediated necroptosis of OLs after injury, while suppressed myelin and axon loss in the white matter after SCI. We then explored the reason and mechanism of increased number of rescued OLs after SCI, and we found that quercetin alleviated macrophages/microglia polarized to M1 phenotype, while promoted M2 polarization after SCI. The new balance of M1-M2 polarization of macrophages/microglia may form a permissive environment allowing for OL survival. Our in vitro study provided direct evidence that quercetin prevented necroptosis of OLs induced by M1 macrophages/microglia.

As numerous intact demyelinated axons are observed after SCI [39], there is an urgent need to rescue myelin sheath. It was reported that p75^NTR^-mediated apoptosis of OLs can be induced by proNGF produced in activated macrophages/microglia after SCI [40, 41]. However, this acute apoptosis of OLs could not explain the chronic, delayed demyelination after SCI [42], suggesting that other types of OLs death accounted for the delayed demyelination. In our previous studies, we demonstrated that necroptosis is a chronic process and necroptosis can occur in OLs after SCI [9, 10]; we thus focused on the effects of quercetin on necroptosis of OLs in the present study. We found that quercetin inhibited necroptosis of OLs and reduced myelin and axonal loss. Further, we examined that whether quercetin could improve OL preservation only or enhance both OL preservation and OL regeneration after SCI. In the previous study, quercetin has been found to promote proliferation and differentiation of OPCs after oxygen/glucose deprivation-induced injury in vitro [43], which is a direct effect of quercetin on OPCs. However, in this study, no significant difference of OL regeneration between control and quercetin-treated rats was found, and we speculated that the microenvironment is one of the possible reasons for the different results between in vitro and in vivo. Moreover, no significant effect of quercetin on apoptosis of OLs was observed. Thus, in the present study, we concluded that quercetin inhibited necroptosis of OLs without influencing regeneration and apoptosis of OLs.

Spinal cord injury can activate quiescent microglia polarized to M1/M2 phenotypes as well as the recruited macrophages. The distinct phenotype may exert their respective roles in pathological event of SCI. The inflammatory response in SCI is characterized by predominant and prolonged M1 macrophages/microglia polarization [35], which forms a detrimental environment for OL survival. Quercetin, a compound acting as anti-oxidative and anti-inflammatory, has been shown to have neuroprotective effects partially by inhibiting inflammatory response after SCI [16]. In our study, we mainly focused on the effect of quercetin on polarization of macrophage/microglia and the data revealed that quercetin suppressed macrophages/microglia polarization to M1 phenotype, while promoted M2 polarization without affecting the total amount of macrophage/microglia.

After hemorrhagic brain injury, reduced M1polarization of microglia was reported by inhibiting TLR4 [44], and we speculated that the difference between our study and the previous research is that we combined LPS and IFNγ to treat cells. It is also known that activation of STAT1 and NF-κB signaling pathways can skew macrophages/microglia toward the M1 phenotype [45]. In this study, we found that quercetin suppressed macrophages/microglia polarized to M1 phenotype through inhibition of STAT1 and NF-κB pathway, which was consistent with the previous results from BV2 cell lines [46]. Nevertheless, the specific mechanism for inhibition of M1 polarization by quercetin requires further study.

Time window for administration of agents is pivotal to therapeutic effect [47]. Although the previous study showed that neuroprotection can be found when quercetin was administered twice daily for a period of either 3 or 10 days [19], we selected the latter because the M1 macrophages/microglia-mediated inflammatory response is a chronic and long-lasting period [35]. In addition, in our preliminary experiment, quercetin alone was administrated to normal rats without SCI, and no effect was found.

Since the functions of microglia and macrophages are similar but not exactly the same [48, 49], a major challenge after SCI is to distinguish microglia and recruited macrophages to determine their phenotype and function. In the present study, we investigated the effects of quercetin on polarization of microglia/macrophages, and one general limitation of our research is that these two distinct cells had not been distinguished. It was reported that application of genetic reporter mice and flow cytometry are the commonly used method to distinguish host microglia and recruited macrophages after injury [50–52]. In the present study, we applied Sprague-Dawley rats instead of genetic mice. Data of cytometry analysis from previous studies showed that CD11b^+^, CD45^low^, Ly6G^−^, and Ly6C^−^ represent the vast majority (> 95%) of microglia after SCI [52, 53]. Therefore, we tried our best to differentiate the host microglia and recruited macrophages by flow cytometry, but we failed to get data due to our technical restrictions. However, this important issue is worthy to be studied in future.

## Conclusion

This study demonstrated that quercetin treatment alleviated necroptosis of OLs at least in part by inhibiting M1 macrophages/microglia polarization after SCI. Its beneficial effects can be potentially useful as a therapeutic agent for clinical SCI.

## Supplementary information

**Additional file 1: Figure S1.** The proportion of apoptotic and necroptotic OLs and the effect of quercetin on apoptosis of OLs. a The co-staining of Cleaved Caspase-3/MLKL/CC1 at different time points after SCI. Scale bar = 50 μm. b-c Quantification of the proportion of apoptotic and necroptotic OLs at different time points after SCI. d-e Quantification and immunostaining of Cleaved Caspase-3 and CC1 in quercetin treated or SCI + saline control rats at 10 dpi. Note that no significant effect of quercetin on apoptosis of OLs. Scale bar = 30 μm. All data are expressed as mean ± SEM. Differences among groups were determined with unpaired two-tailed t test (e). *N* = 6 in each group.

**Additional file 2: Figure S2.** The area that the quantification of Arginase1-positive macrophages/microglia was performed.

## Data Availability

All data generated or analyzed in this study are included in this published article and its additional files.

## Abbreviations

BBB: Basso-Beattie-Bresnahan
GFAP: Glial fibrillary acidic protein
IL: Interleukin
LFB: Luxol fast blue
MLKL: Mixed lineage kinase domain-like protein
OLs: Oligodendrocytes
OPCs: Oligodendrocyte precursor cells
PI: Propidium iodide
PVDF: Polyvinylidene difluoride
qRT-PCR: Quantitative reverse transcription polymerase chain reaction
RHI: Rump-height Index
RIP3: Receptor-interacting serine-threonine kinase 3
RIPA: Radio-immunoprecipitation assay
ROS: Reactive oxygen species
SCI: Spinal cord injury
TLR: Toll-like receptor
