# Supplementary figures and images for: Quercetin prevents necroptosis of oligodendrocytes by inhibiting macrophages/microglia polarization to M1 phenotype after spinal cord injury in rats
Source: J Neuroinflammation. 2019 Nov 7;16:206. doi: 10.1186/s12974-019-1613-2 (PMC6839267; doi:10.1186/s12974-019-1613-2)

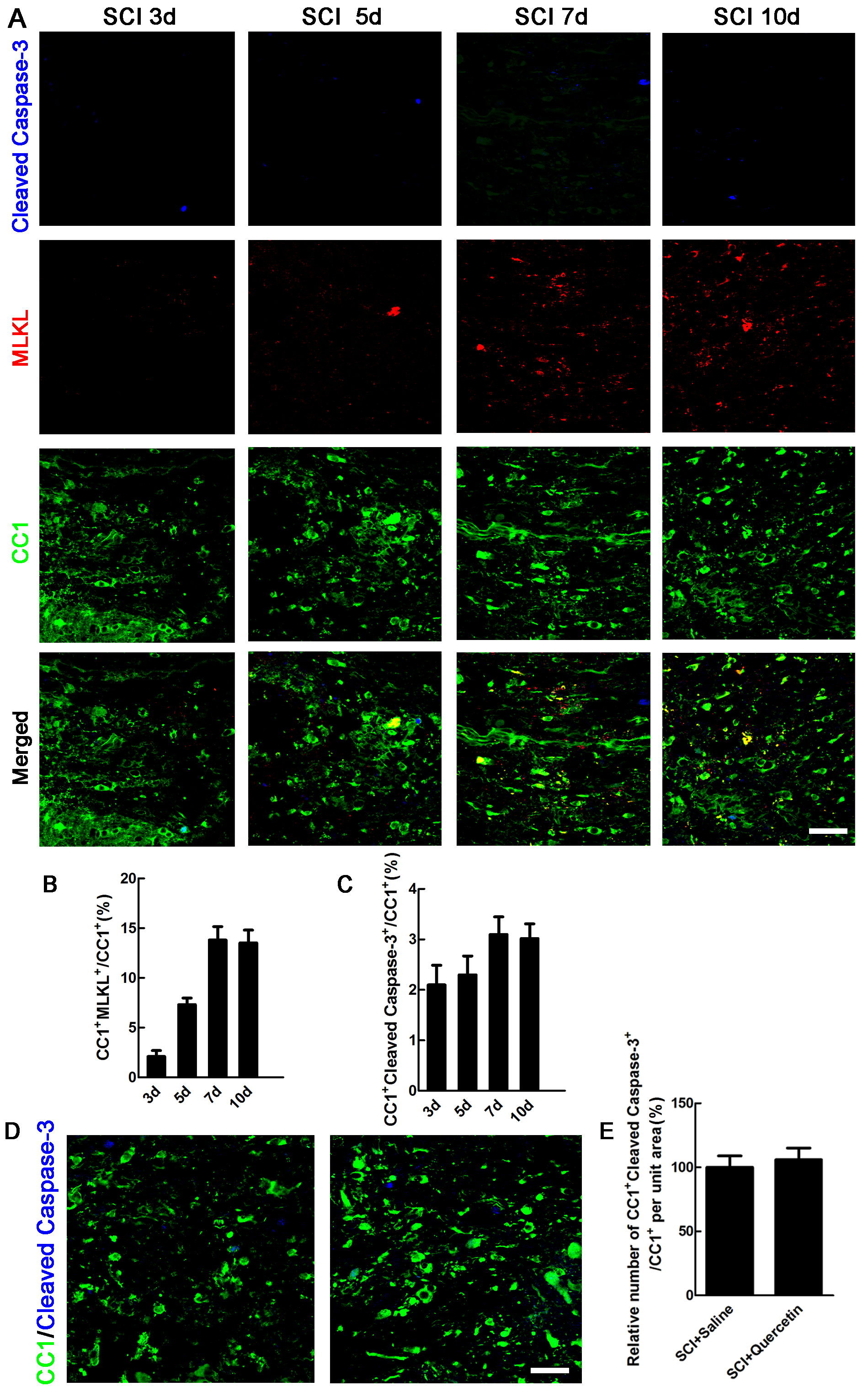

Supplement: Supplementary file 1 — Additional file 1: Figure S1. The proportion of apoptotic and necroptotic OLs and the effect of quercetin on apoptosis of OLs. a The co-staining of Cleaved Caspase-3/MLKL/CC1 at different time points after SCI. Scale bar = 50 μm. b-c Quantification of the proportion of apoptotic and necroptotic OLs at different time points after SCI. d-e Quantification and immunostaining of Cleaved Caspase-3 and CC1 in quercetin treated or SCI + saline control rats at 10 dpi. Note that no significant effect of quercetin on apoptosis of OLs. Scale bar = 30 μm. All data are expressed as mean ± SEM. Differences among groups were determined with unpaired two-tailed t test (e). N = 6 in each group. [file 12974_2019_1613_MOESM1_ESM.tif]

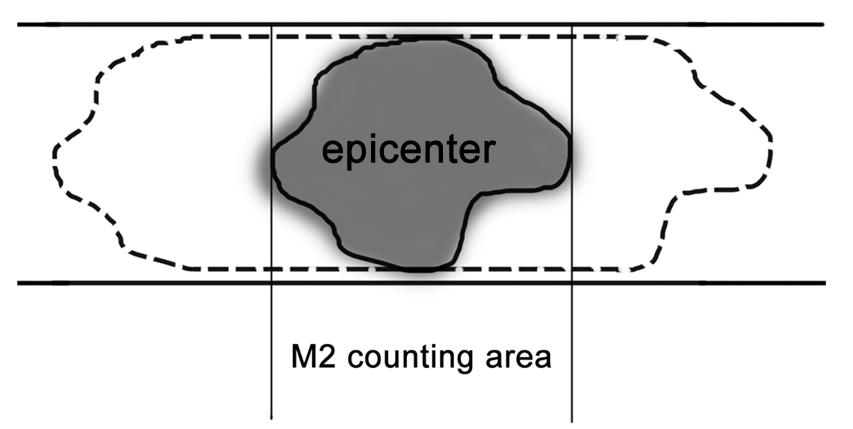

Supplement: Supplementary file 2 — Additional file 2: Figure S2. The area that the quantification of Arginase1-positive macrophages/microglia was performed. [file 12974_2019_1613_MOESM2_ESM.tif]
